# mTOR-Inhibition and COVID-19 in Kidney Transplant Recipients: Focus on Pulmonary Fibrosis

**DOI:** 10.3389/fphar.2021.710543

**Published:** 2021-08-23

**Authors:** Simona Granata, Pierluigi Carratù, Giovanni Stallone, Gianluigi Zaza

**Affiliations:** ^1^ Renal Unit, Department of Medicine, University-Hospital of Verona, Verona, Italy; ^2^ Division of Internal Medicine, Clinica Medica “A. Murri”, Department of Biomedical Sciences and Human Oncology, “Aldo Moro” University of Bari, Bari, Italy; ^3^ Nephrology, Dialysis and Transplantation Unit, Department of Medical and Surgical Science, University of Foggia, Foggia, Italy

**Keywords:** mTOR-inhibitors, COVID-19, pulmonary fibrosis, kidney transplantation, SARS-CoV-2, everolimus, sirolimus

## Abstract

Kidney transplant recipients are at high risk of developing severe COVID-19 due to the coexistence of several transplant-related comorbidities (e.g., cardiovascular disease, diabetes) and chronic immunosuppression. As a consequence, a large part of SARS-CoV-2 infected patients have been managed with a reduction of immunosuppression. The mTOR-I, together with antimetabolites, have been often discontinued in order to minimize the risk of pulmonary toxicity and to antagonize pharmacological interaction with antiviral/anti-inflammatory drugs. However, at our opinion, this therapeutic strategy, although justified in kidney transplant recipients with severe COVID-19, should be carefully evaluated in asymptomatic/paucisymptomatic patients in order to avoid the onset of acute allograft rejections, to potentially exploit the mTOR-I antiviral properties, to reduce proliferation of conventional T lymphocytes (which could mitigate the cytokine storm) and to preserve Treg growth/activity which could reduce the risk of progression to severe disease. In this review, we discuss the current literature regarding the therapeutic potential of mTOR-Is in kidney transplant recipients with COVID-19 with a focus on pulmonary fibrosis.

## Coronavirus Disease 2019 and Pulmonary Fibrosis

Over the last few months, a large number of kidney transplant recipients worldwide have been diagnosed with COVID-19 (Elias et al., 2020; Kataria et al., 2020), and most of them have required intensive care unit (ICU) admission. The reported mortality rate for this patients’ population ranged between 20 and 30% (Akalin et al., 2020; Alberici et al., 2020; Banerjee et al., 2020; Cravedi et al., 2020; Elias et al., 2020; Fernández-Ruiz et al., 2020; Pereira et al., 2020

Main reasons for the dramatic negative clinical impact of COVID-19 pandemic on this fragile cohort of patients is the coexistence of suppression of the immune system by anti-rejection drugs (Banerjee et al., 2020; Cravedi et al., 2020; Pereira et al., 2020) and the occurrence of several comorbidities (Cravedi et al., 2020). Although cardiovascular diseases and diabetes (Li et al., 2020; Muniyappa and Gubbi, 2020) have been identified as major risk factors for worse outcomes from COVID-19 in kidney transplant patients (similar to non-transplant patients), also pulmonary disorders (e.g., chronic obstructive pulmonary disease, interstitial pulmonary fibrosis and chronic sequels of pulmonary bacterial and/or viral infections) have been associated with a significant increased risk of severe disease and death (Drake et al., 2020; Esposito et al., 2020; Higham et al., 2020).

Recent reports have suggested that pulmonary fibrosis associated with severe acute respiratory syndrome coronavirus 2 (SARS-CoV-2) infection may be triggered by both viral- and immune-mediated mechanisms (Liu et al., 2020a) and exacerbated by the acute respiratory distress syndrome (ARDS), occurring in approximately 40% of patients with COVID-19 (Wu et al., 2020). ARDS is characterized by a diffuse alveolar damage with an acute inflammatory exudative process and the release of huge amounts of inflammatory cytokines (Huang et al., 2020) followed by oxidative stress (Chernyak et al., 2020), an organizing phase and an excessive deposition of collagen and extracellular matrix components (Vasarmidi et al., 2020). Additionally, in this condition, SARS-CoV-2 infection in alveolar epithelial cells may provoke an infiltration of immune cells into the lung and an innate immunity activation (Alon et al., 2021).

Adaptive immunity is also activated in COVID-19 patients mainly by antigen presenting dendritic cells, which produce large amounts of cytokines, including interleukin (IL)-6, IL-1β and tumor necrosis factor-alpha (TNF-α) and migrate to the regional lymph nodes to present viral antigen to naïve T cells, pushing their differentiation and migration into affected tissue (Gubernatorova et al., 2020). Among these, cytokines such as transforming growth factor-beta1 (TGF-β1) (Fernandez and Eickelberg, 2012), vascular endothelial growth factor (VEGF) (Barratt et al., 2018), IL-6 (Buonaguro et al., 2020), and TNF-α (Vasarmidi et al., 2020) play a vital role in pulmonary fibrosis.

More than 40% of recovered COVID-19 patients developed pulmonary fibrosis (Li et al., 2021; Zou et al., 2021) and pulmonary impairment also persists after recovery (Mo et al., 2020; Ahmad Alhiyari et al., 2021). The disease duration may impact this condition (approximately 61% of patients with a disease duration greater than 3 weeks developed an important lung fibrosis after ARDS) (Thille et al., 2013; George et al., 2020). This complication occurred more frequently in older, co-morbid patients (mainly with systemic hypertension, diabetes, cardiovascular disease, obesity) who recovered after ICU stay and receiving mechanical ventilation. Additionally, smoking, chronic alcoholism (Ojo et al., 2020) and laboratory findings (lymphopenia, leukocytosis, and elevated lactate dehydrogenase) could predispose individuals to severe lung injury leading to an increased risk of mortality or pulmonary fibrosis in survivors (Liu et al., 2020b).

In kidney transplant recipients, then, immunosuppressive agents may have an impact in pulmonary fibrosis (Gross et al., 1997). In particular, kidney transplant recipients treated with mammalian target of rapamycin inhibitors (mTOR-Is), particularly at high dosages, may develop pulmonary fibrosis (Pham et al., 2004; Weiner et al., 2007; Errasti et al., 2010; Xu et al., 2015; Tomei et al., 2016; Granata et al., 2018).

## Impact of mTOR-is on Pulmonary Fibrosis in Kidney Transplant Recipients: What we Have learned From the Pre-COVID-19 Period

Numerous clinical data have reported an incidence of pulmonary complications in mTOR-I-treated kidney transplant recipients of 2–11%, with the onset of symptoms until 5 years after the initiation of sirolimus or everolimus therapy (Pham et al., 2004; Champion et al., 2006; Weiner et al., 2007; Alexandru et al., 2008; Rodríguez-Moreno et al., 2009; Errasti et al., 2010; Bertolini et al., 2011). There are several lung manifestations including lymphocytic interstitial pneumonitis, lymphocytic alveolitis, bronchiolitis obliterans with organizing pneumonia, focal pulmonary fibrosis, diffuse alveolar hemorrhage or a combination thereof (Morelon et al., 2001; Vlahakis et al., 2004; Vandewiele et al., 2010). This clinical variability and the absence of specific signs and symptoms do not facilitate diagnosis (Molas-Ferrer et al., 2013). Radiographic tests, computed tomography (CT) and bronchoalveolar lavage (BAL), even if often unspecific, are useful diagnostic tools. In some cases, it is also required a lung biopsy that may reveal different histological patterns including the intra-alveolar non-necrotizing epithelioid granuloma, lymphocytic interstitial inflammation and a focal pattern of organizing pneumonia (Kirby et al., 2012).

This pulmonary toxicity seems to be dose-dependent since clinical and radiologic improvement has been observed in a large number of kidney transplant recipients after mTOR-Is dose reduction (Pham et al., 2004; Errasti et al., 2010).

The pathogenic mechanism of mTOR-Is induced pulmonary toxicity is unknown but epithelial to mesenchymal transition (EMT) may have an important role (Zhang et al., 2016). During EMT, epithelial cells lose apical-basal polarity and cell-cell junctions and gain some mesenchymal traits of migration, invasion, and ability to produce extracellular matrix (ECM) (Kalluri and Weinberg, 2009). High dosages of mTOR-I may activate this complex biological process. A massive mTORC1 inhibition, may lead to a down-regulation of S6K and a subsequent hyper-activation of mTORC2 that, sustaining the phosphorylation of AKT at S473, could induce a feedback loop that stimulates PI3K-AKT signaling activating the cellular/molecular machinery leading to fibrosis (Wan et al., 2007; Breuleux et al., 2009; Masola et al., 2013).

Additionally, being EMT regulated by several signaling factors such as TGF-β, epidermal growth factor (EGF), fibroblast growth factor (FGF), IL-1, connective tissue growth factor (CTGF), insulin-like growth factor-2 (IGF-2), nuclear factor-kB (NF-kB) and Wnt (Nieto et al., 2016), pro-fibrotic mTOR-I-associated effects may be enhanced by concomitant pathological conditions including viral and bacterial infections.

Contrarily, other studies have reported that mTOR-I, whether administrated at low dosage, may exert anti-fibrotic effects (Huber et al., 2011; Kurdián et al., 2012; Zaza et al., 2014). Pontrelli et al., have described that rapamycin, reducing plasminogen activator inhibitor (PAI)-1 expression, was able to decrease extracellular matrix deposition in all renal compartments of patients with chronic allograft nephropathy (Pontrelli et al., 2008).

Therefore, based on these results, we encourage transplant clinicians, whenever possible, to prescribe low dose of everolimus/sirolimus in kidney transplant recipients in order to maximize therapeutic efficacy (also anti-fibrotic) and minimize the risk of developing this complication.

## Some Potential Positive Effects of mTOR-I in COVID-19 Patients With a Focus on Pulmonary Fibrosis

The PI3K/AKT/mTOR pathway has been shown to be targeted by various viruses, including influenza virus, herpesvirus, hepatitis C virus and adenovirus (Moody et al., 2005; Sodhi et al., 2006; Bose et al., 2012; Le Sage et al., 2016), and recent studies have clearly reported its activation also during SARS-CoV-2 infection (Appelberg et al., 2020; Lokhande and Devarajan, 2021). The viruses subvert the mTOR pathway to sustain protein synthesis, cell survival and promote virus replication (Moody et al., 2005). mTOR is an evolutionarily conserved serine/threonine kinase, component of two multi-subunit complexes, mTORC1 and mTORC2. Activated mTORC1 induces metabolic effects such as mRNA translation, ribosome biogenesis, protein synthesis, mitochondrial metabolism, and adipogenesis. mTORC2 promotes cell survival, regulates the actin cytoskeleton, ion transport, and cell growth (Dowling et al., 2010). Thus, targeting this pathway might reduce SARS-CoV-2 pathogenicity.

This effect has been also observed in rapamycin-treated cells infected with MERS-CoV and 1918 influenza A virus (Kindrachuk et al., 2015; Ranadheera et al., 2018). Likewise in patients with severe H1N1 pneumonia, early adjuvant treatment with rapamycin and corticosteroids was associated with a rapid virus clearance and a significant clinical improvement (Wang et al., 2014).

In addition, as reported by Garcia et al., nanomolar concentrations of berzosertib, AZD2014 and Torin-2 may exert important anti-SARS-CoV-2 effects by targeting PI3K/AKT/mTOR pathway (Garcia et al., 2021).

Contrarily, an *in vitro* study performed in a human hepato cellular carcinoma cell line demonstrated that rapamycin and Torin-1 failed to block viral infection (Appelberg et al., 2020). However, Akt inhibitor MK-2206, probably by stabilization of mTORC1, showed significant inhibition of viral replication.

Furthermore, the therapeutic potential of mTOR-Is in SARS-CoV-2 infection could also be linked to their immunomodulatory properties.

mTOR pathway, in fact, has a central role in B and T cells development/proliferation. In B cells, mTOR inhibition, through the down-regulation of the transcription factor BCL6, may inhibit germinal center formation (Raybuck et al., 2018) and the proliferation of germinal center B cells thereby hindering development of memory B cells and long-lived plasma cells (Ye et al., 2017). In contrast rapamycin seems to have a minimal effect during the differentiation of germinal center B cells into long-lived plasma cells as well as on the maintenance of already-differentiated long-lived plasma cells (Ye et al., 2017). This effect could influence the response to vaccination in mTOR-I treated kidney transplant recipients.

Moreover, rapamycin, downregulating the expression of activation-induced cytidine deaminase (AID), could also decrease antibody class-switch recombination altering the pattern of immunoglobulin (Ig) G and IgM specificities (Zhang et al., 2013). It has been speculated that this effect may lead to a reduction of the early-stage antibodies crossreactivity against SARS-CoV-2 and to a decrease of the antibody dependant enhancement (LDE). Both these conditions may antagonize the onset and development of severe symptoms (Zheng et al., 2020).

Specific deletion of mTOR in T cells, then, might impair their differentiation in Th1, Th2 or Th17 effector cells (by a direct down-regulation of STAT and other lineage specific transcription factors (Delgoffe et al., 2009)) and induce a significant enhancement of regulatory T cells (Treg) (Battaglia et al., 2005).

At the same time, these agents may have immunostimulatory effects on memory CD8^+^ (Araki et al., 2009) and CD4^+^ T cells (Ye et al., 2017) by promoting the enhancement of memory precursor effector cells that could differentiate into long-lived memory cells (Araki et al., 2009; Ye et al., 2017).

Additionally, mTOR-I when given at the early onset of the cytokine storm phase can hinder the IL-6 pathway and NLRP3 inflammasome-dependent release of IL-1β, thus preventing the progression to severe forms of COVID-19 (Omarjee et al., 2020).

mTOR activation can also increase the activity of anti-inflammatory cytokine IL-10 and inhibit the proinflammatory cytokine TNF-α (Weichhart et al., 2015). Therefore, it is unquestionable that mTOR-Is could act as a double edge sword in patients with COVID-19 (Ghasemnejad-Berenji, 2021) and a correct use of this medication may have a “yin or yang” clinical effects. The ongoing trials (NCT04341675, NCT04461340, NCT04948203) that evaluates the effects of sirolimus treatment in hospitalized COVID-19 patients will provide more information in the next future.

NCT04341675 compares sirolimus (6 mg on day 1 followed by 2 mg daily for the next 13 days or until hospital discharge, whatever happens sooner) versus placebo in hospitalized patients with severe COVID-19. The primary outcome is death or progression to respiratory failure requiring advanced respiratory at day 28.

NCT04461340 asseses the efficacy and safety of sirolimus as an adjuvant agent to the standard treatment protocol against COVID-19. It is a single-blinded randomized study in which participants are randomly assigned to sirolimus (oral dose of 6 mg on day 1 followed by 2 mg daily for 9 days) plus national standard of care therapy against COVID-19 or only national standard of care therapy against COVID-19.

Interestingly, the trial NCT04948203 evaluates the efficacy of sirolimus in preventing post-COVID-19 pulmonary fibrosis. The hospitalized patients with <10% pulmonary fibrosis, evaluated by CT at admission, are divided in three groups according to different dose regimens of sirolimus (0.5, 1 or 2 mg orally daily) for 14 days. Pulmonary fibrosis is, then, evaluated by CT scan after 12 weeks.

## MTOR-I and the Need to Reduce Dosage in COVID-19 Positive Kidney Transplant Recipients

In COVID-19 kidney transplant recipients, at the moment, we do not have enough evidence to support the hypothesis that mTOR-I may antagonize recovery or promote pulmonary complications and contrasting results have been obtained in observational studies (Cravedi et al., 2020; Alberici et al., 2020; Fernández-Ruiz et al., 2020; Caillard et al., 2020; Coll et al., 2021; Favà et al., 2020; Hilbrands et al., 2020; Pérez-Sáez et al., 2020; Salto-Alejandre et al., 2021; Søfteland et al., 2021; Bossini et al., 2020; Crespo et al., 2020; Rodriguez-Cubillo et al., 2020; Meziyerh et al., 2020; Guillen et al., 2020; Zhang et al., 2020; Lauterio et al., 2020; Tanaka et al., 2020; Heron et al., 2021; Nair et al., 2020; Devresse et al., 2020; Maritati et al., 2020; Trujillo et al., 2020; Lubetzky et al., 2020) (Table 1). In studies with large cohort of kidney transplant recipients approximately 10–15% of them were treated with mTOR-Is and more than 50% of these patients stopped this treatment after hospital admission. However, this did not impact the clinical outcomes. Expert opinions suggested to discontinue this drug category in patients tested positive for COVID-19 with or without clinical or radiological evidence of lung disease (Vistoli et al., 2020). This choice could be due to the pulmonary toxicity associated with mTOR-Is (Meziyerh et al., 2020) or to a possible interaction between mTOR-Is and antiviral drugs commonly used in COVID-19 patients (Maggiore et al., 2020). The co-administration of hydroxychloroquine and chloroquine with mTOR-I (all CYP3A4 inhibitors) may theoretically increase their blood concentrations with the development of potential adverse effects/toxicities (including QT-prolongation) (Mirjalili et al., 2020). This condition has been also described in patients treated with Lopinavir/Ritonavir, protease inhibitor largely used in the treatment of human immunodeficiency virus (HIV) and 50–90% reduction in dose of sirolimus and discontinuation of everolimus hase been proposed (Barau et al., 2009; Meziyerh et al., 2020).

**TABLE 1:** Management of mTOR-I therapy and outcomes in kidney transplant recipients with COVID-19

Drug	N° of patients in mTOR-I	Management of mTOR-I	Clinical outcome	Reference
mTOR-I^a^	29/243 admitted to hospital	During hospitalization mTOR-Is were withdrawn in 18/29 (62.1%)	8 developed nonsevere COVID-19 and 10 developed severe COVID-19 200 pts survived (22 were in mTOR-I); 43 died (7 were in mTOR-I) No significant association between clinical outcome and mTOR-I	Caillard et al. (2020)
mTOR-I^a^	131/778 SOT and HSCT recipients (423 kidney transplant recipients)	49 no adjustment; 13 dose reduction; 69 withdrawal	31 pts died (8 in the group of no adjustment, 1 in the dose reduction group and 22 after withdrawal). 47 pts developed ARDS (13 in the group of no adjustment, 1 in the dose reduction group and 33 after withdrawal) No outcome differences were found according to the type of baseline immunosuppression	Coll et al. (2021)
mTOR-I^a^	20/104 (19.3%)	Withdrawn in 12 patients (60%)	76 survived (21.6% were in mTOR-I) and 28 died (3.9% were in mTOR-I) 57 pts developed ARDS (20% were in mTOR-I) 47 pts did not develop ARDS (31.9% were in mTOR-I) No relationship between type of immunosuppression modification and mortality.	Favà et al. (2020)
mTOR-I^a^	14% of 305 (34 pts not admitted and 271 admitted to hospital)	86% no adjustment; 3% dose reduction; 11% withdrawal	207 survived (86% no adjustment; 3% dose reduction; 11% withdrawal) 64 died (87% no adjustment; 0 dose reduction; 13% withdrawal) 213 pts in ICU (14% were in mTOR-I) and 58 pts not ICU (19% were in mTOR-I)	Hilbrands et al. (2020)
mTOR-I^a^	14/80 (17.5%)	26 (33.8%) only MMF or mTOR-I withdrawal 43 (55.8%) both CNI and MMF or mTOR-I withdrawal	54 survived (18.5% were in mTOR-I) 26 died (15.4% were in mTOR-I) In the group of pts with only MMF or mTOR-I withdrawal 15 survived and 11 died In the group of pts with both CNI and MMF or mTOR-I withdrawal 31 survived and 12 died	Pérez-Sáez et al. (2020)
SRL/EVE	49/210 SOT (23.3%) 108 kidney transplant recipients	mTOR-I was decreased or stopped in 35/49 (71.4%)	26 with favorable outcome (full recovery and discharged or stable clinical condition) and 9 with unfavorable outcome (ICU admission and/or death)	Salto-Alejandre et al. (2021)
mTOR-I^a^	14/230 SOT (6.1%) 162 kidney transplant recipients	mTOR-I was decreased or stopped in 2/14	9 inpatients and 5 outpatients 2 patients in mTOR-I died	Søfteland et al. (2021)
mTOR-I^a^	6/53 (11.3%)	Immunosuppression was discontinued in patients required hospital admission	5 admitted to hospital, 1 outpatient. No reported association between mTOR-I treatment and risk of developing ARDS or death	Bossini et al. (2020)
mTOR-I^a^	94/414 (22.7%)	Continued	18 died, 76 recovered. No association between mTOR-I treatment and death	Crespo et al. (2020)
RAPA/EVE	8/29 (27.6%)	Withdrawn	7 patients discharged (2 developed AKI and 1 required ICU admission) 1 patient died	Rodriguez-Cubillo et al. (2020)
EVE	11/144	In most cases reduced or discontinued	6 survived, 5 nonsurvived	Cravedi et al. (2020)

a Drug not specified.

However, although a withdrawal of immunosuppression may have positive effects by restoring the host immunity, it could expose patients to a high risk of acute rejection with negative clinical and psychological impact.

Therefore, mTOR-Is discontinuation should be reserved to kidney transplant recipients with severe COVID-19, while it should be, if possible, avoid in asymptomatic/paucisymptomatic patients in order to do not increase their risk to develop an immune-mediated graft impairment and to take advantage of some potential antiviral proprieties of these agents.

Several clinical trials have reported a reduced rate of Cytomegalovirus and BKV infections in kidney transplant recipients treated with mTOR-Is alone or associated to low dosages of calcineurin inhibitors (CNI) compared to those in standard dose CNI regimen (Tedesco Silva et al., 2010; Brennan et al., 2011; Mallat et al., 2017; Tedesco-Silva et al., 2019; Hauser et al., 2021). The exact mechanism behind this protection is not clear. Compelling data suggest an antivirial role of mTOR-I by blocking cellular proliferation and impairing pathways critical for infection, signaling, and replication (Liacini et al., 2010; Clippinger et al., 2011). In addition, mTOR-I may have a direct anti-viral activity by increasing the percentage of multifunctional virus-specific CD4 T cells (Hauser et al., 2021).

Furthermore, at the moment, the impact of the co-treatment of mTOR-I with other drugs frequently employed in the treatment of COVID-19 kidney transplant recipients (including corticosteroids, anti-inflammatory agents, monoclonal antibodies) has been only partially elucidated. We can only suppose that, being mTOR a central kinase of the cellular metabolism, its inhibition may have consequence on the pharmacological effects of these agents. As reported by Weichhart et al., corticosteroids, inducing the expression of REDD1, may potentiate the pharmacological inhibition of the mTOR pathway. This is in line with previous data obtained in patients affected by H1N1 influenza virus (Weichhart et al., 2011). Additionally, the inhibition of mTOR, preventing the immune hyperactivation of the signal *via* the STAT3 pathway may reduce the expression of receptors for IL-6 and IL-6 production (Terrazzano et al., 2020), that may influence the pharmacological effects of Tocilizumab.

All the above mentioned effects need to be analyzed in specific research project involving organ transplant recipients.

## SARS-CoV-2 Vaccine in Kidney Transplant Recipients

Solid organ transplant candidates and recipients are identified as a priority population for COVID-19 vaccines, given the higher risks associated with immunosuppressed status. Currently, vaccines employable for transplant recipients are BNT162b2 (Pfizer-BioNTech) and mRNA-1273 (Moderna).

However, data regarding safety, immunogenicity, and efficacy in these patients are scarce. Some evidence indicates that solid organ transplant recipients who receive mRNA-based vaccines have low immunization rates (Benotmane et al., 2021; Boyarsky et al., 2021; Danthu et al., 2021; Grupper et al., 2021; Husain et al., 2021; Korth et al., 2021; Sattler et al., 2021) and also in patients with full dose vaccination has reported the development of COVID-19 (Caillard et al., 2021; Tsapepas et al., 2021). When quantitative titers were available, they were frequently below the median titer in immunocompetent patients. Moreover, Rincon-Arevalo et al., have recently described markedly diminished generation of antigen-specific B cells, especially, plasmablasts and memory B cells in kidney transplant recipients (Rincon-Arevalo et al., 2021).

Factors associated with negative humoral response to vaccine were older age, high-dose corticosteroids treatments, maintenance with triple immunosuppressive medications and a regimen that includes anti-metabolite (Boyarsky et al., 2021; Grupper et al., 2021; Husain et al., 2021). The effect of mTOR-I on COVID-19 vaccine is controversial, with some studies reporting a more favorable humoral response (Benotmane et al., 2021; Cucchiari et al., 2021) and other that obtained opposing results (Rozen-Zvi et al., 2021) or no differences in immunosuppressive drugs between kidney transplant recipients tested positive and negative for SARS-CoV-2 IgG (Korth et al., 2021).

Previous studies evaluating the response to vaccination in kidney transplant recipients reported that everolimus or sirolimus are associated with a significant rise in the antigen-specific IgG antibody level after pneumococcal, tetanus and influenza vaccines (Willcocks et al., 2007; Struijk et al., 2010). This could be due to the increment of CD8^+^ effector memory T cells obtained by mTOR-I (Araki et al., 2009; Turner et al., 2011).

However, being these studies not COVID-19 specific and contrasting, we cannot draw definite conclusion on the effects of these drugs on COVID-19 vaccine response. The result of ongoing studies on this topic will help in future to better define this relationship.

## Conclusions and Perspectives

The rapid spread of COVID-19 has pushed physicians to make clinical decisions by the principle of maximizing benefits for the largest number of patients. However, the optimal medical management of kidney transplant recipients with SARS-CoV-2 infection has not yet been established.

The most common approach is the withdrawal of immunosuppressive drugs (including mTOR-ihibitors) in these patients to potentiate their immunocompetence and minimize the risk of clinical complications of severe COVID-19.

However, at our opinion, in kidney transplant recipients in mTOR-Is-based immunosuppressive therapy, this “discontinuation strategy” should be reserved for patients with severe COVID-19. Instead, in asymptomatic patients or those with mild COVID-19 symptoms, a “wait and see approach” or a reduction of the dosage of these agents may be useful to minimize the risk of acute allograft rejection development and to exploit their potential anti-viral and anti-fibrotic effects. The reduction of the dosage may partially restore the host immunity facilitating the disease recovery, antagonize/mitigate the onset of cytokine storm, and preserve Treg growth and activity, which could reduce the progression to severe COVID-19.

Moreover, additional clinical studies aimed to evaluate the impact of mTOR-I on the vaccines and to assess the efficacy and safety of the use of mTOR-I alone or in combination with other new anti-fibrotic agents in kidney transplant recipients with COVID-19 are necessary to allow a more efficient treatment of the acute clinical phase and facilitate the recovery from post-acute COVID-19. Indeed, while most people with COVID-19 recover completely within a few weeks, some patients experience lasting symptoms (fatigue, shortness of breath, cough, joint pain, depression, muscle pain, headache, intermittent fever) that can continue for weeks or even months after initial recovery.

Finally, we believe that molecular biology (particularly omics techniques) may represent powerful methods that could help kidney transplant clinicians to discover new therapeutic strategies for SARS-CoV-2 infection, to select new biomolecular targets and to personalize its treatments (Zaza et al., 2015).

## Abbreviations

ARDS, acute respiratory distress syndrome; EVE, everolimus; ICU, intensive care unit; RAPA, rapamycin; SRL, sirolimus; SOT, solid organ transplantation; HSCT, hematopoietic stem cell transplantation; MMF, mycophenolate mofetil.
